# Serious game prototype for nurses on nipple-areolar lesions resulting from fungal infections in breastfeeding

**DOI:** 10.1371/journal.pone.0341137

**Published:** 2026-03-06

**Authors:** Lays Pinheiro de Medeiros, Luana Souza Freitas, Anna Alice Carmo Gonçalves, Iasmin Freitas Bessa, Rhayssa de Oliveira e Araújo, Isabelle Katherinne Fernandes Costa

**Affiliations:** 1 Januário Cicco Maternity School, Natal, Rio Grande do Norte, Brazil; 2 Nursing department - Federal University of Rio Grande do Norte, Natal, Rio Grande do Norte, Brazil; Link Campus University, ITALY

## Abstract

**Background:**

Despite the advantages, breastfeeding can be a difficult and painful process, especially due to the occurrence of nipple-areolar lesions due to fungal infection. Objective: Develop and validate a breastfeeding software program using a game to be used as an educational tool.

**Methodology:**

An educational software program, the serious game “AleitaGame”, was developed through applied, methodological, quantitative and cross-sectional research. Clinical cases were constructed through a Scoping Review and the media resources and content were chosen. The software, the narrative, the clinical aspects and the context were then validated by experts.

**Results:**

The construction of the clinical case presented and the scenario of a nipple-areolar lesion resulting from a fungal infection was based on 40 scientific documents. The product was then evaluated by two groups of judges.

**Conclusion:**

The study allowed the development and validation of the prototype of serious game on nipple-areolar lesions resulting from fungal infections. The game covers historical content, anamnesis and relevant clinical parameters, the game received good evaluation scores from the judges and it is expected that at the end the player will recognize a nipple-areolar injury, identify signs and symptoms and carry out treatment, monitoring and prevention.

## Introduction

Breastfeeding provides great benefits for the child, as a more complete food supplying all nutritional needs and favoring health promotion throughout their life [1]. For women, the act of breastfeeding also configures multiple gains by being a protective factor of their physical and psychological health and improving self-confidence and connection with the baby for the performance of the role of mother [2].

Despite the advantages, the process can be difficult and painful, due to the various reasons that lead the woman to discontinue breastfeeding [1]. The nipple-areolar complex is responsible for the outflow of milk from the breast, and the lesions caused in it originate pain and early weaning and are caused by multiple factors. One of the causes is fungal infection by *Candida albicans*, manifested by: erythema, peeling, color change of the nipple and/or areola, pain, itching and burning sensation [3–7]. In order to avoid the development of nipple-areolar lesions, nurses need to pay attention to the care in the breastfeeding process, developing strategies for health education of the population. To this end, training and continuous updating of these professionals become indispensable through permanent education actions for qualified and problem-solving assistance [6].

With the advent of health technology, serious games are characterized as a game that, beyond entertainment, provides education. [8,9].

The use of a serious game for teaching subsidizes the learning caused by the strong motivation and involvement of the player and the interest in learning. Given this, it was questioned: what items should compose a serious game scenario about nipple-areolar lesions resulting from fungal infection during breastfeeding? Are the game screens valid? Are the resources used valid? Are the scenario contents valid?

In order to answer these questions, the objective of this research was to develop and validate a prototype of serious game about nipple-areolar lesions resulting from fungal infection during breastfeeding.

## Method

This research is the product of a doctoral thesis and deals with one of the scenarios of “*AleitaGame*”, a great serious game that encompasses three possibilities of teaching about nipple-areolar lesions resulting from breastfeeding, with etiologies and contexts of care of different lesions. Thus, taking into account the amount and variety of theoretical content, gamification techniques, educational resources and learning objectives, we consider each scenario as an individualized game.

### Type of study

This is a methodological research of quantitative approach and cross-sectional character. The development of educational software (ES) followed the methodological framework of Benitti, Seara and Schlindwen [10], from the following stages: design, development, completion and feasibility [10]. The prototype was developed in the second stage.

### Study site

Study conducted in Natal, in the state of Rio Grande do Norte, northeastern Brazil, in 2021. Natal has four maternity hospitals, most of which are certified as Baby-Friendly Hospitals. In 2024, 8,168 births were recorded in the city.

### Study variables

The variables in this study are divided into three: one refers to the game development process, which refers to the type of content included in the game, and the validation process, which includes information about the profile of the research judges, such as age, gender, length of professional experience, qualifications, among others. There are also variables related to the game’s evaluation, such as content agreement and questions about satisfaction, concentration, and engagement in the game.

The first stage establishes the ES guidelines from the definition of learning objectives and organizes them into computational requirements [10], being performed by professionals in the areas of computing and education, represented by the nurses, researchers, themselves. They were responsible for discussing the learning objectives that guided the design of software subsidizing the definition of computational requirements, which were described and developed by professionals in the area of computing through private partnership, with own financing.

The second stage, the elaboration, covers activities of implementation, evaluation and validation of the software. Its purpose is to create a functional prototype of educational software. To this end, activities of specification of increment for initial specification were developed; the prototype was built with attractive interface and attention to usability; and there was the evaluation from appreciation by expert judges who suggest improvements in the content, usability, functionality and performance [10].

After defining the aspects of the game and its learning objectives, an instrument was completed to organize the information.

The virtual game takes place in the scenario of a Basic Health Unit, more specifically, in a nursing office. The items as well as the educational resources present in the prototype were defined from a literature review of the Scoping Review type, and professional experience of the authors. The record of this review is stored in the OSF REGISTRIES, and can be accessed through the link: https://osf.io/s5bwk. The gamification strategies were selected from the learning objectives determined before their development.

With this information, the prototype of the fungal infection scenario was built in close relationship with the developers of serious games and, after the adjustments made by the researchers, the prototype was submitted for evaluation by two groups of expert judges.

### Definition of participants and inclusion criteria

The game in its initial version was submitted to content evaluation by judges expert in breastfeeding who met the following criteria: be a nurse, or have the International Board Certified Lactation Consultant (IBCLC) certification or directly assist lactating women for at least five years. Or be a nurse responsible for the Human Milk Bank working directly in the care of lactating women for at least five years. This experience time criterion reflects the requirements for certification as an IBCLC, in which the candidate must have training in health sciences, a total of 1,000 hours of proven clinical practice in breastfeeding and 90 hours of specific training in lactation, thus meeting the requirements to register for the theoretical test organized by the IBCLC Examiners.

Moreover, the game was assessed regarding technical and pedagogical issues by professionals in the field of technology and education, including people who had at least graduation and with practical experience in programming educational software; and/or have carried out research in the area of development and/or application of educational software.

### Instruments used and data collection

The data collection of the studies of the scope review and ordering and disposition of the information for composition of the scenario were performed from an instrument based on studies that addressed the construction of clinical cases. This instrument contained the necessary steps to create a clinical case with its descriptions, gamification techniques and learning objectives, according to Bloom’s taxonomy.

Participants for this study were identified from the list of IBCLC-certified professionals in Brazil, available at <https://aleitamento.com.br/ > . They were then contacted via email to invite them to participate in the study. If they accepted, the consent form, along with the survey form (prepared in Google Forms), was sent, and data collection took place through Google Meet meetings. Data collection time was between 30 and 45 minutes.

The data collection instrument for content evaluation was made available through a link from Google Forms subdivided into three sessions: data on the professional profile of the research judge, evaluation of the game content regarding the narrative, clinical and contextual aspects addressed in the scenarios and evaluation of the prototype as an educational strategy through the items of the adapted Egameflow instrument [11].

This instrument was judged on a scale of one (1) to seven (7) in 20 categories, namely: whether the game caught my attention; I am not distracted from the tasks I should concentrate; I enjoy the game without getting bored or anxious; the difficulty is adequate; the skills increase with as the game progresses; motivation for improvement of skills; feeling of control of the menu; the game allows the recovery of mistakes made; feeling of using any strategies; knowing the next step of the game; general objectives presented at the beginning of the game; understanding of learning objectives through the game; receiving feedback from progress in the game; receiving information about status (level or score); forgetting time, things around and day-to-day problems while playing; involvement with the game; improvement of knowledge from the game; application of knowledge in the game; desire to know more about the content presented, and an open question to describe what was possible to learn from the prototype.

The data collection instrument for the second group of judges (technology and education) was made available through a link from Google Forms, subdivided into three sessions: data on the professional profile of the judge of the research and evaluation of the technical and pedagogical aspects in two subsections containing each of the items of the following instruments: Learning Object Review Instrument (LORI) and adapted Egameflow [11,12]. The evaluation process for all judges was conducted remotely, through online meetings on the Google Meet platform, in which the researcher was able to register suggestions for improvements beyond the written in the evaluation form.

LORI considers aspects of content quality, alignment of learning goals, feedback and adaptation, motivation, presentation design, usability of interaction and accessibility, classifying as poor (1), regular (2), good (3), very good (4) and excellent (5).

Recruiting started 10/01/2021, finished 15/01/2021.

### Treatment and Data analysis

The data were collected through Google Forms and subsequently extracted and organized in an Excel spreadsheet. Scoping was based on tabulated data and descriptively analyzed according to information found in the studies selected for the sample. Descriptive statistical analysis was used to develop the serious game, with the Content Validity Index (CVI) for the content evaluation section, assessing the narrative, clinical aspects, and context, and the mean score of responses obtained by the Egameflow and LORI instruments. The results are presented in tables and textual descriptions.

The content evaluation was done by 6 nurses and the technical aspects were evaluated by 3 technology professionals.

### Ethical aspects

In accordance with the ethical and scientific rigor for research developed with human beings, the study was evaluated by the Research Ethics Committee of UFRN identified by CAAE 15860819.0.0000.5537, obtaining a favorable opinion with number 3,552,016. The participants received the Informed Consent Form (ICF) in the virtual format, requiring the signature of the document. Written consent was obtained.

There were no consequences after withdrawal from the study, there were no direct or indirect benefits to the participants, the risks of the research included participant fatigue in participating in the meeting, evaluating the game and completing the instruments, but these were minimized by scheduling the time of data collection in a timely and convenient manner for the participant, and the information obtained was kept confidential.

The game has the certificate of registration of computer program to guarantee intellectual property with process number: BR512021003084−2.

## Results

The construction of the clinical case presented and the scenario of nipple-areolar injury due to fungal infection was based on 40 scientific documents, which were selected from the sample of the Scoping Review, considering only those that described the lesions related to fungal infections, and experience of the researchers. From this, the content and media resources of the serious game presented by texts and podcast were selected. Chart 1 shows the description of the contents and teaching resources used (Table 1).

**Table 1:** Description of the content and teaching resources used. Natal/RN, 2021.

Context	Teaching resource
Clinical manifestation of fungal infection in the child’s oral cavity	Real photo-type image
Clinical manifestations of fungal infection in the nipple of postpartum women	Real photo-type image
History	Fictitious medical record
Anamnesis	Narrative in dialogue format
Relevant clinical parameters	Multiple choice question
Counseling	Podcast
Factors related to fungal infection in the mother-child binomial	Text
Harms of using pacifiers	Text
Subjective descriptions related to fungal infection in the nipples	Text
Most appropriate conduct for the case	Multiple choice question

Source: Research.

After construction, there was evaluation by the two groups of judges. The first group, composed of six specialists in the field of breastfeeding, had as profile: female (100.0%), mean age of 54.6 years, with average time of graduation in Nursing of 31 years, 66.6% have the IBCLC certification, one worked in a Child-Friendly Hospital (16.7%) and one was Technical Responsible (TR) in the Brazilian Network of Human Milk Banks (HMBn-BR) (16.7%), 50% had a master’s degree and the other half had specialization, as for complementary training, most (50%) had as master’s object of study the thematic of maternal breastfeeding, and two of these also had specialization in maternal breastfeeding, one (16.7%) had specialization in Obstetrics, one (16.7%) in Pediatrics and Neonatology and one (16.7%) did not enter complementary training.

Most nurses worked in Rio Grande do Sul (50%), 33.4% in Rio Grande do Norte and 16.7% in Paraná. All nurses worked in the care of postpartum women with nipple-areolar lesions and the average time of operation was 22.3 years.

The evaluation of the first group of judges presented CVI 1 (maximum score) in the three criteria evaluated. Judges could point out necessary adjustments and had the option to submit additional comments if they wished. Chart 2 below details the judges’ assessment, comments and suggestions (Table 2).

**Table 2:** Assessment, comments and suggestions from content judges.

Evaluative criteria		CVI*
Narrative		1
Clinical Aspects		1
Context		1
**Is the case adequate to teach this topic about care for nipple-areolar injuries?**		**n (%)**
Yes		5 (83.3)
Yes, but requires small changes		1 (16.7)
No		0 (0.0)
**JUDGES’ COMMENTS**		
“Nipple trauma x fungal infection (I don’t think it’s correct to present the infection as trauma). Fungal infection does not cause loss of integrity of the nipple tissue. The baby may not have a lesion in the mouth. Observe the presence of diaper rash on the baby.”		
“Often in practical experience, the baby does not have the lesions in the mouth so visible, they may be very small or not found at all.”		
“Often the fungal infection is not so evident, neither in the mother’s breast nor in the baby’s mouth. I think this is an aspect that should be highlighted. “		
“Explore more symptoms in the mother and baby (baby’s perineum, vaginal infection in the mother, time elapsed, etc.)”		
“I consider it very relevant, due to the frequency of this type of injury in the late postpartum period.”		
**JUDGES’ SUGGESTIONS**		
**Judge’s suggestion**	**Change executed**	**Description/Justification**
Add information about the possibility that the clinical presentation of the fungal infection may not be as evident as presented in the scenario, and may even not present objective signs, both in the child and in the mother.	Yes	This information was added in textual format to the scenario.
Add other areas of investigation into fungal infections in mothers and children.	Yes	This information was added in textual format to the scenario.

Natal/RN, 2021.

*Content Validity Index.

Source: Research.

The suggestions were considered adequate and all requests were accepted and adjustments were made in the final version of the game.

The second group of judges, which evaluated the technical and pedagogical aspects, was composed of three professionals in the area of technology and education, two (66.7%) male, all (100%) with master’s degree, two (66.7%) were researchers in the field of educational games and one (33.3%) had training to act as a developer of games focused on education. Judges had been working and/or studying in the area for an average of 4.3 years.

The evaluation of these judges, through LORI, pointed out better average scores in the variables: presentation design (4.7), content quality (4.6) and alignment of learning goals (4.3). The usability of interaction received a mean score of 3.6, motivation, feedback and adaptation received a score of 3.3 and accessibility a mean score of 2. Considering 5 as the maximum score, most items reached at least 66% of this parameter.

As for the evaluation of the game by the EGameflow instrument, all judges use technological resources daily, 33.4% of judges reported that they rarely play, 22.2% play every day, 22.2% play occasionally and 22.2% never play.

Regarding the evaluation items, the mean scores of the content judges (group 1) varied between 6 and 7, while the scores of the judges of the technical and pedagogical aspects (group 2) ranged between 3 and 7. The question that got the lowest score in the two groups of judges was as follows: [I forget the day-to-day problems while playing?}, with an average score of 6 for group 1 and an average score of 3 for group 2, The questions that presented higher average scores in both groups were: [I understand the objectives of learning through the game?} and [I get feedback on my progress in the game?} reaching a maximum score of 7 each, by both judges. In the items where there was divergence >1.5 points between the two groups of judges, lack of closeness and interest with the theme of the judges in the area of technology and education can be attributed.

The table containing the detailed information can be accessed in the article: “Virtual Simulation on breastfeeding and nipple-areolar lesions: development and validation of prototype.”

Some suggestions for changes were also made by the judges who evaluated the technical and pedagogical aspects, being accepted and adjusted for the final version of the game. The observations concerned: adding verbs in the infinitive, differentiate colors of text boxes, remove the option to advance while the player is still exploring the scenario, highlight captions, explain in the feedback the wrong items and add the option “try again” in the final feedback of the scenario.

After all the modifications were performed based on the evaluations, the scenario of assistance to nipple-areolar injury due to fungal infection was finalized and moved forward to the stage of completion and feasibility.

**Fig 1:**
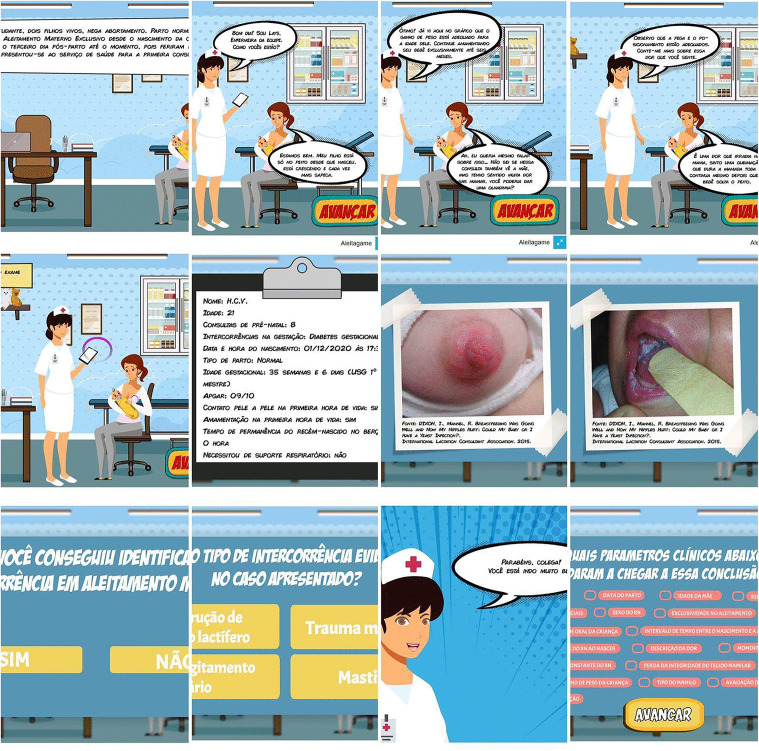
Game interfaces in the scenario of nipple-areolar injury due to fungal infection during breastfeeding. Natal/RN, 2021. Source: Research.

Fig 1 shows a summary with the game interfaces, comprising the learning objectives: recognition of key points of case evaluation, interpretation of favorable and unfavorable clinical aspects for nipple-areolar lesions and demonstration of knowledge by solving problems about the case.

As observed, the game addresses contents of history, anamnesis and relevant clinical parameters, as well as the didactic resources used: fictitious medical record, narrative in dialogue format and multiple-choice question. The gamification features used in these screens were: narrative, levels of integration, feedback and points. The final version of the game can be viewed at: https://www.youtube.com/watch?v=cinf-ux_Fqw

In the end, the player needs to recognize a nipple-areolar lesion, identify signs, symptoms and conditions that determine the diagnosis and know how to perform the treatment, monitoring and prevention of lesion due to fungal infection.

**Fig 2:**
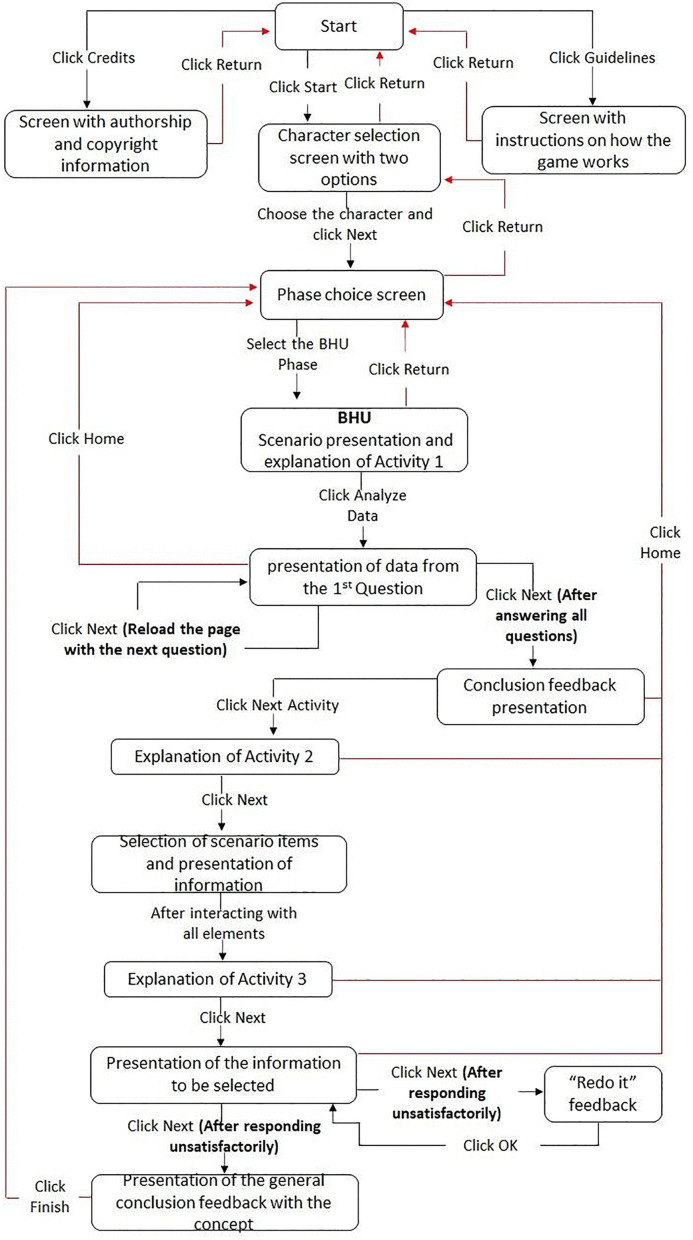
Game interaction flow diagram. Natal/RN, Brazil, 2021. Source: Research.

The flow diagram is detailed in Fig 2 below.

The variables found in the study point to the relevance of this complication that can cause suffering and loss of breastfeeding, interfering with the health and well-being of the mother and baby, clarifying the manifestations as visible or invisible signs and symptoms, as well as highlighting the procedure to treat and prevent the occurrence of the disorder studied.

The knowledge covered in the clinical scenario and the basis of the serious game developed allows the professional to identify and stop any reinfection that may occur, promoting safe breastfeeding and healthy child development.

## Discussion

The basic health units, as structures that provide assistance at the level of primary health care, are great allies and deserve prominence regarding the monitoring of exclusive breastfeeding (EBF) for the period of six months, recommended by the World Health Organization (WHO) [13].

The nurse, as the professional closest to the health care of women and babies, should be able to identify changes that harm the health of both and intervene effectively to solve problems [6].

As identified in the scoping review, nipple-areolar lesions due to fungal infection have great impact on discomfort when breastfeeding and in early weaning, thus, the nurse has a primary function in the detection, treatment and monitoring of cases [7].

In the developed game, it was possible to present certain objective clinical manifestations through real images that had: erythema, peeling, change in the color of the nipple tissue and/or areola and skin with bright appearance; and also by subjective manifestations that, in consensus with the literature, were characterized by: persistent pain during and after breastfeeding, pain that radiates to the breasts (especially after breastfeeding), itching and burning sensation. Clinical presentations are not always evident, which may hinder the diagnosis, delaying treatment and consequently leading to the abandonment of breastfeeding. Both objective and subjective manifestations need to be considered in the clinical evaluation of the professional [14].

Concerning the treatment, the pharmacological treatment is carried out with topical and oral medications. Topical treatment is based on antifungal drugs such as miconazole, ketoconazole and clotrimazole applied to the nipple after breastfeeding, for 10–14 days. Gentian violet (solution 0.25% to 5%) was also used on the nipple twice a day for three days, and the home treatment consisted of the application of vinegar solution (a teaspoon of white vinegar diluted in a cup of tea or water) on the nipples [15].

Authors point to the use of topical mupirocin three times a day for five to seven days and indicate caution with violet due to the risk of lesions in the baby’s oral mucosa [16]. As a topical medication, the ointment or nystatin cream was found to be applied to the nipple three to four times a day, one to two weeks [17].

As for oral treatment, fluconazole was identified with an attack dose of 400 mg and reinforcement of 200 mg per day for 14–21 days, and another study verified the medication with the dosage of two doses of 150 mg, one every 48 hours or 100 mg per day for 10 days [16]

During the evaluation by judges, one of them suggested the inclusion of investigation of other sources of fungal infection in the baby. In addition to the oral cavity, the judge in compliance with the literature pointed out sources of infection such as vulvovaginal candidiasis, infection concomitant to diaper rash and vaginal infection in women [15].

On non-pharmacological care, the game states: “dry treatment: keep nipples dry and airy” and “discourage the use of teats, pacifiers and bottles”. These measures, in line with the scientific literature, should be encouraged to prevent infection. Guidelines such as hand hygiene and washing of fabrics, toys and other objects that may be contaminated can also be included [15].

As for the development and use of the game, several areas have benefited from the use of serious games, for being a dynamic teaching strategy that allows the union of education with attractive interactivity [18]. In the health field, games can train graduated professionals to assist a specific population, give students the opportunity to learn new knowledge or review important aspects on a topic [8,9,19].

It is important to use educational resources based on scientific literature and validated by professionals in the field. In addition, the relevant evaluation with the target audience is highlighted in order to verify the effectiveness and satisfaction of the population benefiting from the knowledge produced. A study carried out in the Northeast of Brazil with professionals who work as preceptors for the Multiprofessional Residency in Maternal and Child Health at a public institution evaluated the effectiveness of the Aleitagame game in terms of theoretical knowledge and satisfaction, including the fungal infection scenario dealt with in this study [20].

The researchers carried out a quasi-experimental, single-group, before-and-after study, in which 43 professionals from 8 professional categories answered a knowledge instrument containing 10 questions in the pre-test, then played the aleitagame and then answered the same knowledge instrument (post-test) and the satisfaction questionnaire containing 19 statements. The authors concluded with their findings that the game increased the population’s knowledge in most of the questions investigated (7 questions out of a total of 10). The knowledge questions about the fungal infection scenario had the following results: the question about the diagnosis of nipple lesions had 30 hits in the pre-test (69.8%) and 38 hits in the post-test (88.4%); the question about guidance for cases of breast candidiasis had the same number of 34 hits (79.1%) in the pre and post-test; and the question about signs and symptoms of breast candidiasis had an increase of 6 hits, from 34 (79.1%) in the pre-test to 40 (93.0%) in the post-test [20].

In evaluating the effectiveness of this study, there was a statistically significant increase in theoretical knowledge with an increase of 0.6 points in the average, and the investigation of satisfaction with the game was considered excellent by at least 86% of those surveyed. Thus, it can be seen that the development game was effective as an educational resource and was satisfactory for most of the professionals surveyed [20].

The authors demonstrated that a serious game developed for COVID-19 safety behavior increased nursing students’ knowledge and confidence. The serious game provides a realistic environment and allows students to practice their knowledge without jeopardizing patient safety, reinforcing learning through the opportunity for multiple playthroughs [21].

A study conducted in Northern Ireland presented nursing students with a game about the influenza vaccine and obtained satisfactory results, with a significant increase in vaccination adherence, knowledge about the vaccine, and the importance of promoting vaccination to the general public and their future patients [22].

Another research report tested the serious game MemoreBox on elderly people in nursing homes and observed improvements in cognitive abilities in the elderly who played regularly when compared to the control group [23].

Researchers in Shanghai, China, conducted a study comparing pre-test, post-test, and retention knowledge among medical students after a six-month intervention. Comparing data from the control group and the intervention group, in which the latter had access to the serious game NEOGAMES, it was observed that in the pre-test, both groups had similar average scores. However, there was a significant improvement in knowledge in the intervention group, with a 3.3-point increase over the control group’s average, and knowledge retention in the intervention group was almost three times better than that of the control group [24].

For meaningful learning, the individual must have an interest in learning, thus, serious games unite fun with education, allowing good performance of the learner. Aspects such as attractive interface and usability deserve attention in the development of health technologies in order to engage the user in the use of the software [25].

Regarding the game developed, called “*Aleitagame*”, it has an innovative character because the literature revealed other games about nipple-areolar lesions, and deserves attention for the awareness of a condition with frequent occurrence, impact and that is occasionally disregarded due to lack of preparation and professional knowledge to act efficiently [26].

Other studies in the area of breastfeeding may be developed from the awareness of the problem exposed, and also other studies of development and evaluation of serious games with different populations.

As limitations, the difficulty in finalizing the other stages of software development stands out, due to the need for application with target audience impossible by the pandemic state. Another limitation is the use only by professionals with nursing degrees, although nursing technicians act in support of breastfeeding, the condition presented in the game requires nursing consultation that is private activity of the nurse. However, it does not prevent technical-level professionals in nursing or other health categories who assist women in breastfeeding from exploring the resources that the game has.

## Conclusion

The study allowed the development and validation of the prototype of serious game on nipple-areolar lesions resulting from fungal infections. The game was developed in conjunction with professionals of technology and health and education, from the national and international literature and the empirical experience of the researcher, and allowed answering which items should compose a serious game scenario about nipple injuries resulting from fungal infection during breastfeeding.

After the development, there was the evaluation with two groups of judges on the content and technical and pedagogical aspects, which provided answers that the screens, resources and content of the scenario developed in the serious game are valid.

From this, it is hoped that the game can help trained and in-training professionals to provide qualified care to their patients, in order to identify nipple-areolar lesions caused by fungi, and know how to treat and teach how to prevent them in order to guarantee the growth and development of the child, well-being for the breastfeeding woman and the health of all.

The final version of the game containing this and other scenarios can be accessed for free through the website: https://aleitagame.github.io/

## Supporting information

S1_File Additional information.
(ZIP)
