# Supplementary material for: Serious game prototype for nurses on nipple-areolar lesions resulting from fungal infections in breastfeeding
Source: PLoS One. 2026 Mar 6;21(3):e0341137. doi: 10.1371/journal.pone.0341137 (PMC12965671; doi:10.1371/journal.pone.0341137)
Supplement: S1_File — Additional information. (ZIP) [file pone.0341137.s001.zip › S1_File_Supporting_Information.docx]

All relevant data are within the paper and its Supporting Information files.
